# On the Conversion of Animal Muscle into a Substance Much Resembling Spermaceti

**Published:** 1797

**Authors:** George Smith Gibbes

**Affiliations:** of Magdalen College, Oxford.


XIII. *On the Converſion of Animal Muſcle into a Subſtance much reſembling Spermaceti. By* George Smith Gibbes, *B. A. of Magdalen College, Oxford.* Vide *Philoſophical Tranſactions of the Royal Society of London, for the Year* 1794, Part. II. *And for the Year* 1795, Part II. 4to. London, 1794—5.

IN a former volume* of this work, ſome account was given of the diſcoveries lately made at Paris, relative to the converſion of certain parts of the human body, under particular circumſtances, after death, into a ſubſtance reſembling ſpermaceti. M. Thouret, to whom we owe the firſt deſcription of this phenomenon, ſeemed diſpoſed to aſcribe it to the extrication of aeriform fluids from the dead body during putrefaction, and to the reaction of thoſe fluids on the body itſelf; but the experiments related by Mr. Gibbes in the two very ingenious papers on this ſubject now be-

* Vol. I. page 186.

fore fore us, ſerve to ſhow that the putrefactive ferment is not at all neceſſary in the formation of the ſubſtance in queſtion.

It is a matter of great curioſity, our author remarks, to obſerve, after any fact has been well aſcertained, how many things might have led to a much earlier inveſtigation of it; particularly ſo, had the writings of many great men been equally examined, with thoſe obſervations which, though apparently very trifling, have often excited general attention. Of this we have a very ſtriking example in the converſion of animal muſcle into a fatty matter, a fact of which Mr. Gibbes has diſcovered traces in the works of two celebrated philoſophers of our own country, Sir Thomas Brown, and Lord Bacon. The former of theſe, in his very learned and curious treatiſe, entitled *Hydriotaphia*, aſſures us that he has found a ſoap-like ſubſtance in an hydropical body. His words are as follow, viz. " In an hydropical body, " ten years buried in a church-yard, we met " with a fat concretion, where the nitre of the " earth, and the ſalt and lixivious liquor of " the body, had coagulated large lumps of fat " into the conſiſtence of the hardeſt Caſtile " ſoap; whereof part remaineth with us:" and

Lord

Lord Bacon, in his work entitled *Sylva Sylvarum*, expreffly fays, " You may turn (almoft) " all flefh into a fatty fubftance, if you take " flefh and cut it into pieces, and put the " pieces into a glafs covered with parchment, " and fo let the glafs ftand fix or feven hours " in boiling water. It may be an experiment " of profit for making greafe or fat for many " ufes; but then it muft be of fuch flefh as is " not edible, as horfes, dogs, bears, foxes, " badgers, &c."

After having feen fome of the matter found in the burial place of the Innocents at Paris, our author concluded that in fome fituations the fame kind of fubftance might be eafily found; accordingly he examined fome of the macerating tubs belonging to anatomical fchools in town, and he found that in moft of them the flefh was nearly changed into this kind of fat. By the indulgence of Dr. Pegge, the anatomical profeffor in Oxford, he was permitted to examine the receptacle in which the bodies are depofited, after he has finifhed lecturing on them. This place is a hole dug in the ground to the depth of about thirteen or fourteen feet, and, to remove all offenfive fmell, a little ftream is turned through it. Mr. Gibbes found, found, on firſt looking into it, that the fleſh was quite white, and on drawing up the firſt piece, he found it changed in the manner before deſcribed. From this place he has procured, he tells us, at leaſt twelve pounds weight of a ſubſtance equal in every reſpect to ſpermaceti.

Having ſeen many ſpecimens of different animals, which had been changed under ſomewhat different circumſtances, that is, where ſome had been buried in dampiſh ground, ſome in wet ground, and ſome even in water itſelf, he began to ſuſpect that the ſame change might be brought about in a ſhorter time, at leaſt that he might determine the time neceſſary for it: with this view a piece of the leaneſt part of a rump of beef was confined in a box full of holes, which being tied to a tree near a river, was ſuffered to float in it. On taking this up from time to time, he perceived that it gradually got whiter and whiter, and at the end of a month it was perfectly, to appearance, changed to a maſs of fatty matter. From ſome circumſtances, he is induced to believe that it is ſooner converted in running water than when it is perfectly at reſt; for while this beef was expoſed to the water in the river, a piece of mutton was was placed in a reſervoir of water, and he perceived, that though the mutton was expoſed for a longer time than the beef, yet that it was not ſo much changed.

Finding that this ſubſtance was ſo formed, and that he could procure large quantities of it, Mr. Gibbes made ſome experiments to purify it; for this purpoſe he took ſeveral pieces of it and melted them, and he found, though they were brought into a cloſer union, yet that the fœtid ſmell was as bad as before. After trying ſome unſucceſsful experiments, it occurred to him, that if he could add a ſubſtance to it which would unite with the offenſive parts, and not with the fat, he might then get it pure; accordingly he poured ſome nitrous acid upon it, which immediately had the deſired effect; a waxy ſmell was perceived, and on ſeparating and melting it, he got it, we are told, nearly pure. The nitrous acid, he obſerves, turns it yellow, but by ſubmitting it to the action of the oxygenated muriatic acid, he has got it quite white and pure.

In the beginning of June, 1793, Mr. Gibbes buried a cow in a place where, from the riſing of a river to ſupply a mill twice a day, it was ſubmitted to the action of running water. On taking taking this cow up in December, he found, that where the water was conſtantly running over it, there it was changed into a fatty ſubſtance; but where the water which had acted on the meat could not paſs off, there a very diſagreeable ſmell was ſenſible, and the fleſh was not ſo much changed. A piece of this cow, that was perfectly lean, was ſtuck through with a ſtick, and faſtened to the bottom of the river; this piece, he aſſures us, was perfectly changed into a fat matter, and had loſt its offenſive ſmell.

Mr. Gibbes adds, that he has brought about this change in a much ſhorter time, in the following manner: he took three lean pieces of mutton, and poured on them the three mineral acids, and he perceived that at the end of three days each was much altered; that in the nitrous acid was much ſoftened, and on ſeparating the acid from it, he found it to be exactly the ſame with that which he had before got from the water; that in the muriatic acid was not ſo much altered; the vitriolic acid had turned the other black.

From theſe experiments our author thinks it appears, that it is not at all neceſſary that the putrefactive fermentation ſhould take place; but, but, on the contrary, that it takes away a great deal of the flesh which might serve for the formation of a greater quantity of this waxy substance.

We come now to Mr. Gibbes's second paper on the same subject. In his first, as we have seen, he observed that the substance procured either by means of water, or of the nitrous acid, appeared to have precisely the same external characters; but, it seems, he has since observed a difference between that obtained from quadrupeds, and that which is procured from the human subject: the former, we are told, seems not disposed to crystallize, while the latter assumes a very beautiful and regular crystalline appearance.

After melting some of the matter procured from human muscle, our author plunged into it a very sensible thermometer, which soon rose to 160°; at 112° the substance began to congeal, and at 110° became so solid, that the thermometer could not be easily taken out.

Mr. Gibbes took some of the spermaceti of the shops, and under the same circumstances plunged the same thermometer into it. It soon rose rofe to 170°; a pellicle was formed at the top of it when at 117°; and it became fo folid at 114°, that the thermometer could not eafily be taken out.

He diffolved a piece of the fubftance, which he had formed by means of water and the nitrous acid, in boiling fpirit of wine; and on cooling this mixture, a great quantity of this waxy matter was feparated in the form of beautiful flakes. He could not procure large cryftals, but the flakes, he obferves, affumed a cryftalline appearance.

He put into an earthen retort fome of this waxy matter, to which he added fome finely powdered charcoal; and on applying a pretty ftrong fire, a fmall quantity of an oily fluid came over, which concreted on cooling; after this came over a great quantity of thick white vapours, which were very fuffocating and offenfive.

Into a copper retort he put a fmall quantity of this matter, and placed it on a common fire; there came over firft a limpid fluid like water, without much fmell; on the addition of more heat, there came over an oily fluid, which foon coagulated, of a firmer confiftence than when put in, and coloured of a beautiful green by by the copper; this laſt circumſtance, Mr. Gibbes obſerves, proves that it contained no ammonia.

Having procured ſome very pure quickſilver, our author took a glaſs, which contained about ten pounds of that fluid, with which he filled it; he inverted it in a baſon which contained the ſame fluid, and then introduced a ſmall piece of lean meat, and alſo a ſmall quantity of water. At the end of about ſix weeks, ſo great a quantity of gas, we are told, was diſengaged as nearly to occupy the whole of the veſſel; and the meat had aſſumed a white appearance.

Since the account given in his former paper of his experiments on the cow, which he had ſubmitted to the action of running water, Mr. Gibbes has obſerved ſome facts relative to the changes which took place, and which he now deſcribes. This cow, as we have ſeen, was placed in a ſituation where the water could come twice every day; and over it was thrown ſome looſe earth. After it had remained ſome time in this place, our author uſed frequently to puſh a ſtick through this earth to the cow; and he obſerved, that every time this was done there came up a great quantity of air, after he had suffered it to remain quiet for a short time. He has since, he tells us, had two horses and another cow placed in a similar situation; and in all of them, it seems, this disengagement of air takes place. This air, he adds, is extremely offensive.

Mr. Gibbes observes, that in the former cow the whole muscular part seemed changed; and that from the substance formed he procured a very large quantity of a waxy substance by means of the nitrous acid. Though the nitrous acid takes off the greatest part of the fœtor from the substance thus formed, yet it gives it, it seems, a yellow colour, which is with difficulty removed, and a peculiar smell, evidently similar to the smell of the acid employed, which mere washing and the addition of alkalies will not entirely remove.

Our author observes that his father, who has been indefatigable in his attempts to whiten this substance, has found a method by which it may be rendered very pure and beautiful, though not so white as the spermaceti of the shops. The process was as follows: the cow, which had lain in the water for a year and an half, was taken up, and the whole muscular part was found perfectly changed into a white matter; matter; this was broken into ſmall pieces, and expoſed to the action of the ſun and air for a conſiderable length of time. By theſe means it loſt a great deal of its ſmell, and ſeemed to acquire a firmer conſiſtence. The appearance of this ſubſtance was ſomewhat ſingular; for on breaking it, little filaments were ſeen running in every direction, exactly ſimilar to the cellular ſubſtance between the muſcular fibres. Theſe pieces were then beaten to a fine powder, and on this powder was poured ſome diluted nitrous acid; after the acid had been on it for about an hour, a froth was formed at the top; the acid was then poured off, and the ſubſtance repeatedly waſhed; it was then melted in hot water, and when it concreted it was of a very beautiful ſtraw-colour, without the leaſt offenſive ſmell: on the contrary, it had the agreeable ſmell of the beſt ſpermaceti.

May not this ſubſtance, our author aſks, be applied as an article of commerce? Great quantities of it, he obſerves, may be obtained. It burns with a fine flame; and dead animals, which at preſent are of little or no uſe, may be changed into it. He regrets that it has not been in his power to aſcertain the preciſe quantity which may be obtained from a given quantity of flesh; but from what he has obtained, he can say that it would be very considerable. The running water carries off a great deal of it; but that, he observes, might be obviated by the addition of strainers. Moreover, he adds, that which is carried off by the water is the purest. The water over the animals, and for some distance round them, he tells us, is covered with a very beautiful pellicle, which is white in general; and sometimes refracts the sun's rays, producing the prismatic colours.

Fish, the author observes, may be also changed; and he recollects to have seen, in some old writer whose name he cannot recollect, an account of something of this kind happening in a whale. The writer states, that after this fish has been putrifying on the shore some time, the people have a secret by which they can procure and purify lumps, similar to the spermaceti which they get in the usual way.

On seeing a body opened some time ago, where there was a great collection of water in the cavity of the thorax, Mr. Gibbes observed that the surface of the lungs was covered with a whitish crust; and he remarked to a friend, that he he thought this cruſt was owing to ſome combinations which had taken place between the lungs, or pleura, and the ſerous fluid effuſed, ſimilar to what he had obſerved between fleſh and water; or that the ſerous fluid had acted on the coagulable matter, and had produced a ſimilar change. He has found a curious fact mentioned by Dr. Cleghorn, which in ſome meaſure ſeems to agree with the obſervation then made. It occurs in that part of his Obſervations on the Diſeaſes of Minorca, (p. 248) where he is ſpeaking of abſceſſes in the lungs, and is as follows: "Theſe abſceſſes had ſometimes emptied themſelves into the cavity of "the thorax, ſo that the lungs floated in purulent ſerum; their external membrane, and "likewiſe the pleura, being greatly thickened, "and converted, as it were, into a white cruſt, "like melted tallow grown cold." In a note Dr. Cleghorn ſays, "I am now doubtful if this "cruſt was the pleura and external coat of the "lungs, changed from a natural ſtate by ſoaking in a purulent fluid; and if it was not "altogether a preternatural ſubſtance, formed "by fluids depoſited on thoſe membranes, and "compacted together by the motion of the "lungs."

Mr. Gibbes obferves, that much has been faid by different writers on the fubject of fecretion, which at one time was fuppofed to depend on fome peculiar property of the living principle; fo that it was thought impoffible to form any fecretion but through the medium of fecreting organs. M. Fourcroy, however, he adds, has contradicted this by the experiments where he forms bile.

Spermaceti is an animal fubftance, fecreted in a particular fpecies of whale, and the fubftance which is formed in the foregoing experiments, as far as our author can judge, agrees with it in every particular.

M. Fourcroy fays, that M. Poulletier de la Salle found a cryftallized inflammable fubftance fimilar to fpermaceti in biliary calculi.

May not the fuety matter in fteatomatous tumours, our author afks, arife from fomething of this kind? And by attending to the various fecretions of the body, by examining their compofition in the healthy and morbid ftates of the fyftem, may we not expect to derive great advantage, particularly when accurate experiments are applied towards the relief of difeafe?

Mr. Gibbes candidly obferves, that fome excufe excuſe may perhaps ſeem neceſſary for the little attention which has been paid to the accurate reſults in the different experiments he has deſcribed; particularly ſo, as the analyſis of every part of the animal body, except the bones, is at preſent ſo incomplete; but he hopes that the time neceſſary for his medical purſuits, and the want of a complete chemical apparatus, will not render the ſimple facts he has related leſs uſeful.

He has not attempted, he tells us, to account for the various phenomena which appear in the experiments, becauſe the facts ſeemed too few to admit of any general concluſion.

